# Supplementary material for: The Human Aspect of Horse Care: How the COVID-19 Pandemic Impacted the Wellbeing of Equestrian Industry Stakeholders
Source: Animals (Basel). 2021 Jul 22;11(8):2163. doi: 10.3390/ani11082163 (PMC8388490; doi:10.3390/ani11082163)
Supplement: Supplementary file 1 [file animals-11-02163-s001.zip › animals-1299009-supplementary.pdf]

Supplementary file 1. Table S1.

**Theme**	**Sub-theme**	**Condensed meaning unit**	**Wellbeing impact**
The ways the industry communicates	Teleconsultation	Positive for clients	Fulfilling domain specific satisfaction
		Better vet-client relationships	Stimulating work activity
		Increased workload - no increased pay	Affecting work-life balance / increasing stress at work
		Bullying behaviours	Affects self-esteem / confidence / increase frustration.
		Too many calls in one day	Affecting work-life balance / increasing stress at work
		Juggling admin with calls	Affecting work-life balance / increasing stress at work
		Hard to judge condition	Non-stimulating / frustrating work activity / stress
		Organisations benefit more than vets	Resentment for organisation / stress
		Vets made decision to switch	Resentment for organisation / stress
	Digital training	Chance to keep up with training	Pursue hobby / sport, skill development
		Maintain horse fitness	Animal healthy, owner satisfied, domain satisfaction
		Engage in routine	Maintain daily routine, familiarity, comfort, life satisfaction
		Spend time outdoors	Relaxation and enjoyment, life satisfaction
		Interact with friends	Social wellbeing
		Enter competition	Pursue hobby / sport, goal setting

		Engage in CPD	Fulfilling domain specific satisfaction
	Social media	Communicated to broad audience	Good for wider community, work activity
		Interact with others online	Social wellbeing
		Share experiences	Fulfilment from interaction / feedback from peers, social wellbeing
		Rapidly share important information	Good for wider community, work satisfaction
		Makes others jealous	Negative for peer-peer relationships
		Aggravates others	Negative for peer-peer relationships
		Possibility to receive judgement from others	Negative for peer-peer relationships, negative for mental health
Reduced contact with horses	Closing yards	Lack of interaction with horse	Loneliness, lack of positive animal feedback
		Change in routine	Psychological impact of unfamiliar routine
		Loss of social circle	Loneliness, lack of positive human feedback
		Missing horse-riding activity / sport	Loneliness, lack of positive human feedback, reduced physical activity
	Fearing for horse health	Will horse be shod	Psychological impact animal perceived as being vulnerable
		Worried around vaccinations	Psychological impact animal perceived as being vulnerable
		Worries around tooth rasping	Psychological impact animal perceived as being vulnerable
		Worried around weight gain	Psychological impact animal perceived as being vulnerable
		Worried for health / colic if they are fed by public	Psychological impact animal perceived as being vulnerable

	Lacking positive interactions	Physical interaction with horse helps day-to-day stress	Human -animal bond strong & perceived as necessary to cope
		Need horse time to decompress	Human -animal bond strong & perceived as necessary to cope
		Spending time with animals helps relieve worry	Human -animal bond strong & perceived as necessary to cope
Prosocial behaviour	Virtual socialising /fund raising	Zoom calls to raise money for BHS	Altruism positive for community, emotional wellbeing improved
		Quiz to interact with others	Strong social bonds form in yard environment
		Maintain relationship with yard groups	Strong social bonds form in yard environment
	Supporting community	Farrier shoeing horses for free	Community values
		Farriers refusing to "steal" clients	Community values
		Creating content for owners to feel "part of something"	Understanding of yard social groups
		Developing novel ways for owners to interact with their horses	Understanding of the importance of human-horse interaction
		Providing social support group for owners.	Strong social bonds form in yard environment
	Valuing time with horses	Little things feel more important now	Importance of practical horse care tasks in mental wellbeing
		People with horses fared better than those without	Fortunate position
		Important to take stock of the journey with horses	Importance of practical horse care tasks in mental wellbeing
		Grooming, walking, caring for horses of higher value now	Importance of practical horse care tasks in mental wellbeing

Table S1 shows a summary of themes, subthemes and the summarised meaning units interpreted from the interview transcripts, as well as the specific wellbeing associated impact relating to the subject raised in the meaning unit.
